# Genotyping of hepatitis C virus by nucleotide sequencing: A robust method for a diagnostic laboratory

**DOI:** 10.1016/j.mex.2018.04.005

**Published:** 2018-04-16

**Authors:** Elina Virtanen, Laura Mannonen, Maija Lappalainen, Eeva Auvinen

**Affiliations:** Department of Virology, University of Helsinki and Helsinki University Hospital Laboratory, Helsinki, Finland

**Keywords:** HCV, hepatitis C virus, LiPA, line probe assay, PCR, polymerase chain reaction, RT, reverse transcription, UTR, untranslated region, HCV genotyping, HCV, Genotype, PCR, Amplification, Sanger, 5’UTR

## Abstract

Hepatitis C virus (HCV) is a globally significant blood-borne agent causing liver diseases, and it has infected over 170 million people worldwide. HCV is a diverse group of RNA viruses currently divided into genotypes 1–7 as well as subtypes. HCV infection can be treated with antiviral drugs, but the HCV genotype has to be determined for optimal selection of treatment strategy. The aim of this study was to set up a sequencing-based HCV genotyping method suitable for the workflow of a diagnostic laboratory. The established method is robust and stable, and it utilizes a one-step reverse transcription and PCR amplification of the 5′ untranslated region (5′UTR) and partial Core region of the HCV genome. Amplification products are sequenced using the standard Sanger method, and the genotype is determined by using a freely accessible web-based genotyping tool. The method was validated at the Helsinki University Hospital Laboratory using 238 previously genotyped serum samples.

• A new one-step RT-PCR method for the amplification of the 5′ untranslated region and partial Core region of hepatitis C virus was established.
• HCV genotype is determined using Sanger sequencing and a freely accessible, easy-to-use web-based genotyping tool.
• The method is robust, reproducible and suitable for diagnostic laboratory workflow, and it requires no costly instrumentation or specialized sequence analysis skills.

## Graphical abstract

**Figure:**
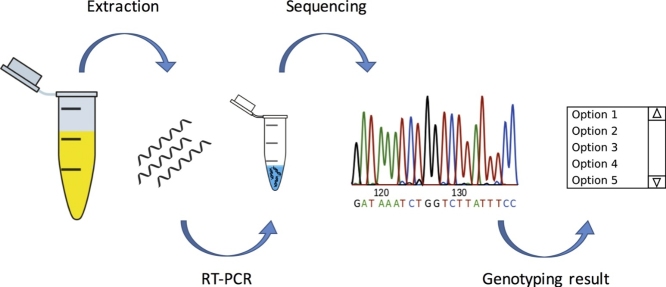

Specifications Table

**Table:** 

Subject area	• *Immunology and Microbiology*
More specific subject area	*Molecular diagnostics of viruses*
Method name	*HCV genotyping*
Name and reference of original method	*Other HCV amplification methods exist, but this method has been independently designed, developed and established.*
Resource availability	Reagents: http://www.thermofisher.com/fi/en/home/references/protocols/nucleic-acid-amplification-and-expression-profiling/pcr-protocol/superscript-3-one-step-rt-pcr-system-with-platinum-taq-high-fidelity.html
	Genotyping tool:
	https://www.ncbi.nlm.nih.gov/projects/genotyping/formpage.cgi

## Method details

The HCV genotyping method described here was developed for a diagnostic laboratory workflow, aiming at a simple and straightforward protocol. A requirement for the method was that all genotypes should be amplified using only one primer pair in a single amplification reaction. The method utilizes the detailed and objective information provided by nucleotide sequencing, but requires no specific analysis skills or costly tools to determine the main HCV genotype.

### RNA extraction and PCR amplification

1) Extract nucleic acids using the automated NucliSENS easyMAG extraction system (bioMérieux, Marcy-l'Étoile, FR). Use 200 μl of serum and Generic 2.0.1 protocol for extraction, and elute nucleic acids into 25 μl of NucliSens Extraction Buffer (BioMerieux). If PCR amplification is not performed directly after extraction, the nucleic acid preparations can be stored in −70 °C.
2) For PCR amplification, use SuperScript III One-Step RT-PCR System with Platinum Taq High Fidelity polymerase (Invitrogen, Thermo Fisher Scientific, Waltham, MA, USA), where reverse transcription (RT) and PCR reactions are run in one reaction.
3) Set up a 25 μl PCR reaction containing 0.9 μM of forward (5′ GTC TAG CCA TGG CGT TAG TAT GAG TG 3′) and reverse (5′ ACA AGT AAA CTC CAC CAA CGA TCT G 3′) primers, 12.5 μl of 2 x reaction mix (Invitrogen), 0.5 μl of SS III RT/Platinum Taq HiFi enzyme mix (Invitrogen) and 3 μl of nucleic acid template.
4) Run the reactions in a standard thermal cycler. In our laboratory the DNA Engine Tetrad®2 Peltier Thermal Cycler (BioRad, Hercules, CA, USA) was used with the following RT-PCR conditions: cDNA synthesis for 30 min at 55 °C, RT-enzyme inactivation for 2 min at 94 °C, 40 cycles of 15 s denaturation at 94 °C, 30 s annealing at 58 °C and 1 min extension at 68 °C, followed by a final extension for 5 min at 68 °C and cooling to 4 °C.
5) The success of amplification can be inspected using gel electrophoresis. In our laboratory 1.8% agarose gels stained with ethidium bromide, or the fast electrophoresis FlashGel System (Lonza, Basel, Switzerland) were used. The expected product length is 374 bp.

### Sanger sequencing and sequence analysis

1) The amplification products should be sequenced in both directions using standard Sanger sequencing and the amplification primers described above. Our samples were sequenced at the Institute of Biotechnology, University of Helsinki.
2) Inspect the retrieved chromatograms by using a standard program, for example Chromas (http://technelysium.com.au/wp/chromas/) or 4peaks (http://nucleobytes.com/4peaks/). Copy the high quality sequence portion with unambiguous individual peaks for analysis. The forward and reverse sequences are analysed separately for each sample without making one consensus sequence.
3) Paste the sequences in FASTA or text format to NCBI’s Internet based genotyping tool available at: http://www.ncbi.nlm.nih.gov/projects/genotyping [1]. The tool compares the inserted sequence to a HCV sequence database and gives a list of best matches with distinct genotype and subtype information arranged according to scoring points, highest scores indicating the best match. Sequences can also be analysed using the Standard Nucleotide BLAST, available at: https://blast.ncbi.nlm.nih.gov/Blast.cgi?PAGE_TYPE=BlastSearch [2].

## Method validation

### Samples

All samples (N = 238) used in the establishment and validation of this method had been previously genotyped at the Helsinki University Hospital Laboratory (HUSLAB), Department of Virology and Immunology, by using the Versant HCV Genotype 2.0 (Siemens Healthcare, Tarrytown, NY, USA) line probe assay (LiPA). Viral loads were quantified for a proportion of samples using the COBAS® AmpliPrep/COBAS® TaqMan® HCV Quantitative Test version 2.0 (Roche Molecular Diagnostics, Pleasanton, CA, USA). After initial analysis the samples had been frozen and stored at −20 °C.

### Selection of primers

For primer design 5′UTR, core/E1 and NS5B regions were selected. For each region 2–4 primer pairs were designed by aligning and comparing reference sequences within the selected regions and by selecting sequences which would share maximal homology between different genotypes. The requirement was that each genotype should be amplified using only one primer pair in a single amplification reaction. For the design process the Basic Local Alignment Search Tool (BLASTN) 2.2.27+ [2] and Primer-BLAST [3] programs were used. The reference sequences were M62321 (genotype 1a), D90208 (1b), D14853 (1c), D00944 (2a), D10988 (2b), D50409 (2c), D17763 (3a), D49374 (3b), Y11604 (4a), Y13184 (5a) and Y12083 (6a). Some primer sequences that had been previously published from our laboratory were utilized as well [4]. All primers (Integrated DNA Technologies, Coralville, IA, USA) were initially tested at annealing temperatures of 50 °C and 55 °C, after which the temperatures were further adjusted at intervals of 3 °C starting from the temperature which produced stronger amplification products for each genotype. All primer sequences and annealing temperatures for each primer pair, as well as success of amplification are presented in Table 1.

**Table 1:** Primers tested for PCR amplification of different HCV genotypes. Nucleotide numbering and product length are according to HCV 1a isolate H77 (GenBank accession number AF009606). Primers amplifying several genotypes are highlighted in bold.

Primer	Start	End	Sequence (5' → 3')	Product length (nt)	Annealing temperature	Genotypes amplified
**5’UTR 1 F**	77	102	GTCTAGCCATGGCGTTAGTATGAGTG	374	58 °C	1a, 1b, 2a, 2b,
**5’UTR 1 R**	450	426	ACAAGTAAACTCCACCAACGATCTG			3a, 4, 5a, 6
5’UTR 2 F	74	91	AGCGTCTAGCCATGGCGT	265	50/55 °C*	Several short
5’UTR 2 R	338	321	GCACGGTCTACGAGACCT			products
5’UTR 3 F	73	90	AAGCGTCTAGCCATGGCG	267	50/55 °C*	Several short
5’UTR 3 R	339	322	TGCACGGTCTACGAGACC			products
5’UTR 4 F	143	159	GTGGTCTGCGGAACCGG	174	50/55 °C*	No amplification
5’UTR 4 R	316	299	GGGCACTCGCAAGCACCC			products
Core/E1 1 F	733	750	CCGACCTCATGGGGTACA	589	55 °C	2a, 3a
Core/E1 1 R	1321	1299	GACCAGTTCATCATCATATCCCA			
Core/E1 2 F	841	859	GGAACCTTCCTGGTTGCTC	481	50/55 °C*	No amplification
Core/E1 2 R	1321	1299	GACCAGTTCATCATCATATCCCA			products
**Core/E1 3 F**	842	869	GAACCTTCCTGGTTGCTCTTTCTCTATC	487	47 °C	1a, 1b, 2a, 3a,
**Core/E1 3 R**	1328	1306	CGTAGGGGACCAGTTCATCATCA			4, 6
NS5B 1 F	7952	7970	CCACATCAACTCCGTGTGG	684	50/55 °C*	2a, (2b, 1a)
NS5B 1 R	8635	8616	CTGGTCATAGCCTCCGTGAA			
NS5B 2 F	8010	8031	ACCATCATGGCCAAGAACGAGG	632	50/55 °C*	No amplification
NS5B 2 R	8641	8620	GAGTACCTGGTCATAGCCTCCG			products
**NS5B 3 F**	8010	8031	ACCATCATGGCCAAGAACGAGG	698	50 °C	2a, 2b, 3a, 4,
**NS5B 3 R**	8707	8682	TTGGAGGAGCATGATGTTATAAGCTC			(1b)
**NS5B 4 F**	7953	7976	CACATCAACTCCGTGTGGAAAGAC	755	52 °C	1a, 1b, 2a, 4,
**NS5B 4 R**	8707	8682	TTGGAGGAGCATGATGTTATAAGCTC			(6)

Genotypes which were inconsistently amplified are given in brackets.

* Primers were tested using two different annealing temperatures which performed equally.

### PCR optimization

For initial primer testing, a set of eight samples representing all main genotypes and most common subtypes (1a, 1b, 2a, 2b, 3a, 4, 5a and 6) was used. Out of all primers, only 5′UTR1F and 5′UTR1R were able to amplify all samples in our testing set, and were thus selected for further optimization. Reaction conditions were adjusted for the following parameters: annealing temperature, primer concentration, magnesium concentration, amount of template and number of amplification cycles. Using the optimized PCR conditions presented above, a total of 238 serum samples were amplified (Table 2). Strong amplification products were generally obtained from samples whose viral loads were over 10 000 IU/ml. Distinct but weaker products were obtained from samples whose known viral loads were 1020–9810 IU/ml. Samples which remained negative in gel had viral loads of 52–2550 IU/ml. Amplification with the established method did not directly correlate with viral load. This may be due to long storage time and multiple freeze-thaw cycles of the samples. The sensitivity of the method was found adequate for genotyping of patient samples, whose viral loads are generally between 10^4^–10^7^ IU/mL.

**Table 2:** Success of HCV genotyping using the established method.

LiPA genotype^a^	1	2	3	4	5	6	1a/4	2b/1a	Unknown^b^	NC^c^	Total
Samples amplified	78	37	100	13	4	1	2	1	1	1	238
Product in gel	71	32	96	12	4	1	2	1	0	0	219
No product in gel	7	5	4	1	0	0	0	0	1	1	19
Samples sequenced	64	30	92	7	4	1	1	1	1	0	201
Retrieved genotype results	64	28	91	7	4	1	1	1	0	0	197

a Versant HCV Genotype 2.0 (Siemens Healthcare, Tarrytown, NY, USA) line probe assay.

b Genotype could not be identified using the LiPA method.

c Negative control, a patient sample tested negative for HCV.

### Sequencing

Altogether 201 amplification products were sequenced (Table 2), including nine samples where no amplification product was observed in agarose gel. In total, 197 genotyping results were obtained, including five gel-negative samples. The four samples where no genotyping result was obtained were all gel-negative amplification reactions. The length of the obtained sequences was approximately 300 bp for the majority of samples. The sequence based genotyping results were consistent with LiPA results except for three samples, where a different result at the main genotype level was obtained. For one sample the LiPA method yielded an equivocal result of genotype 2 while sequences in both directions matched genotype 3a. For the second sample both sequences indicated genotype 1b whereas LiPA identified genotype 5a. For the third equivocal sample no amplification product was seen in gel, and only a short reverse sequence indicating genotype 2k was derived, while the LiPA result indicated genotype 1. For two additional samples, two alternative genotypes were reported from LiPA analysis: 2b/1a and 1a/4. The forward and reverse sequences from both of these samples clearly matched genotype 1.

## Conclusions

5′UTR sequences suit well for main HCV genotype determination and in certain cases for subtype determination as well. However, because of its conserved nature, the 5′UTR cannot be applied to reliably distinguish genotype 1 subtypes. The established genotyping method is straightforward and robust, fits well to the diagnostic laboratory workflow, and does not require costly instrumentation or specialized sequence analysis skills.

## References

[1] Rozanov M., Plikat U., Chappey C., Kochergin A., Tatusova T. (2004). A web-based genotyping resource for viral sequences. Nucleic Acids Res..

[2] Altschul S.F., Madden T.L., Schaffer A.A., Zhang J., Zhang Z., Miller W., Lipman D.J. (1997). Gapped BLAST and PSI-BLAST: a new generation of protein database search programs. Nucleic Acids Res..

[3] Ye J., Coulouris G., Zaretskaya I., Cutcutache I., Rozen S., Madden T.L. (2012). Primer-BLAST: a tool to design target-specific primers for polymerase chain reaction. BMC Bioinform..

[4] Pohjanpelto P., Lappalainen M., Widell A., Asikainen K., Paunio M. (1996). Hepatitis C genotypes in Finland determined by RFLP. Clin. Diagn. Virol..

